# Effects of early factor XIII replacement in postpartum hae morrhage: study protocol for a multicentre, open-label, randomised, controlled, investigator-initiated trial

**DOI:** 10.1136/bmjopen-2025-100262

**Published:** 2025-05-08

**Authors:** Christian Haslinger, Torsten Hothorn, Verena Bossung, Stylianos Kalimeris, Elisabetta Ranieri, Nicole Ochsenbein-Koelble, Wolfgang Korte

**Affiliations:** 1 Department of Obstetrics, University Hospital Zurich, Zürich, ZH, Switzerland; 2 University of Zurich, Faculty of Medicine, Zürich, ZH, Switzerland; 3 Institut für Epidemiologie, Biostatistik und Prävention, Universität Zürich, Zürich, Switzerland; 4 Zentrum Für Labormedizin (ZLM), St. Gallen, Switzerland

**Keywords:** Postpartum Period, Randomized Controlled Trial, Bleeding disorders & coagulopathies, Pregnant Women

## Abstract

**Introduction:**

The primary objective of this trial is to evaluate the effect of replenishing coagulation factor XIII (FXIII) in women with postpartum haemorrhage (PPH) on measured blood loss (MBL). Based on earlier research, we hypothesise that the administration of FXIII leads to a significant reduction in postpartum blood loss.

**Methods and analysis:**

This is a randomised, controlled trial that will allocate eligible patients in the event of a PPH and after receiving tranexamic acid either to the treatment group, receiving FXIII, or to the control group (standard of care). The primary endpoint is the MBL within 24 hours using a standardised method. For the primary analysis, estimation of the OR under a proportional odds assumption is conducted simultaneously for all possible cut-off points. A corresponding estimate, along with a two-sided 95% CI and two-sided p value against the null hypothesis OR=1, is obtained from the Continuous Outcome Logistic Regression model. More than 7000 patients will be screened in order to include a total of 988 eligible patients into the trial. Secondary outcomes include the rate of adverse maternal outcomes related to PPH, the rate of women breastfeeding after PPH and the safety of the administration of FXIII in women with PPH. Dynamics of blood coagulation factors in women with PPH and their association with MBL will be assessed in specific centres. A preliminary overview on costs and potential savings through early treatment of PPH with FXIII is included in the analysis as well as a patient and public involvement report, asking for patients’ personal experience during PPH in the main study centre.

**Ethics and dissemination:**

Ethics approval was granted by the central ethics committee (Kantonale Ethikkommission Zürich/Switzerland) on 16 June 2024, reference number: BASEC 2024-00374. Results will be disseminated via open-access publication in a relevant medical journal.

**Trial registration number:**

ClinicalTrials.gov ID NCT06481995.

**STRENGTHS AND LIMITATIONS OF THIS STUDY**

- This multicentre trial involving the largest perinatal centres in Switzerland examines a new approach in the treatment of postpartum haemorrhage (PPH), namely the early administration of blood coagulation factor XIII in case of increased postpartum bleeding.
- Participating sites will use a standardised method for blood loss measurement after vaginal deliveries, ensuring the accurate documentation of the primary outcome, which is postpartum blood loss.
- This trial will significantly contribute to the comprehension of coagulopathy in the context of PPH by blood sample analysis before and after delivery.
- The necessary recruitment is considered ambitious due to the high number of screening failures caused by the unpredictability of PPH, which will lead to a significant workload in participating sites in a clinical 24/7 emergency setting.
- Replenishment of FXIII during the early stage of PPH cannot be compared with placebo since the costs of production, packaging and subsequent approval of a placebo product would exceed the resources available for this investigator-initiated study.

## Introduction

 Postpartum haemorrhage (PPH) is the main reason for maternal mortality and morbidity.^1 2^ PPH, defined by the WHO as blood loss of 500 mL or more within 24 hours after delivery, causes about 30% of maternal deaths worldwide morbidity.^1^,^4^ The internationally observed trend towards increased PPH-related morbidity and mortality is disturbing and demands new strategies in the prevention and treatment of PPH.^5^,^9^

Although the most frequent causes for severe PPH are believed to be uterine atony or placenta-related problems (placenta accreta spectrum, retained placenta or placental tissue), virtually all cases of severe PPH lead to a disorder of the coagulation system, which itself aggravates bleeding.^10 11^

At the moment, most guidelines on coagulation management and expert opinions focus on the replenishment of coagulation factor I (fibrinogen),^12^,^14^ although three out of three randomised controlled trials with early or pre-emptive administration of fibrinogen during PPH were negative.^15^,^17^

Based on earlier research,^18^,^20^ it was hypothesised that coagulation factor XIII (FXIII) might also play a significant role in women with increased postpartum blood loss because of its role in the establishment of blood clot stability and fibrinolytic resistance. In fact, a prospective diagnostic study involving 1300 parturient women at the University Hospital Zurich showed that prepartum FXIII activity had a very strong association to postpartum blood loss, stronger than all other prepartum parameters evaluated.^21^ In consequence, this nationwide, multicentre, open-label, randomised controlled trial in major perinatal centres across Switzerland will be conducted to assess whether the early administration of FXIII reduces postpartum blood loss and PPH-related complications. FXIII will be replenished at an early stage of PPH, at 700 mL of blood loss after vaginal delivery and continued bleeding, after having received 1 g of tranexamic acid (TXA) at a blood loss of 500 mL. If a woman continues to experience bleeding despite receiving 1 g TXA, hyperfibrinolysis can be considered not to be the sole issue, and a further treatment modality such as FXIII might be beneficial. The combination of the results mentioned above, together with the knowledge that FXIII activity, is significantly reduced in pregnant women during the second and third trimesters (compared with women in the first trimester of pregnancy and a control group),^22^ and the administration of FXIII in a randomised, controlled trial is considered duly justified.

The article summarises the protocol of the SWIss Factor XIII Trial, also known as the SWIFT Trial. In the light of increasing PPH rates and negative randomised controlled trials for the administration of fibrinogen, new ways of thinking are inevitable to reduce the burden of PPH. Although the realisation of a multicentre, randomised controlled trial in parturient women in an emergency situation remains ambitious, it is inevitable to test the hypothesis that early replenishment of FXIII reduces postpartum blood loss in women with PPH. While a few clinical sites use FXIII regularly today in later stages of PPH management,^23^ there is no randomised, controlled trial providing evidence-based knowledge on the efficiency of FXIII, even though its use is already to be considered according to the updated guidelines from Germany, Austria and Switzerland. The SWIFT project will provide scientific evidence on the question of early substitution of FXIII in postpartum haemorrhage.

## Methods and analysis

### Study design and setting

Nine clinical sites will participate in this trial. Together, they represent the largest perinatal centres in Switzerland, including all Swiss university hospitals. Sites were involved early on for the development of the protocol. Emphasis was put on designing a study process that is compatible with the national PPH guideline as well as routine processes at each site.

Plasma-derived FXIII (Fibrogammin, CSL Behring) is used in accordance with the indication and dosages described in the summary of products characteristics. Based on the Swiss laws for clinical research in humans, the present trial is a category A trial, meaning a low-risk trial.

### Eligibility of patients

**Figure 1:**
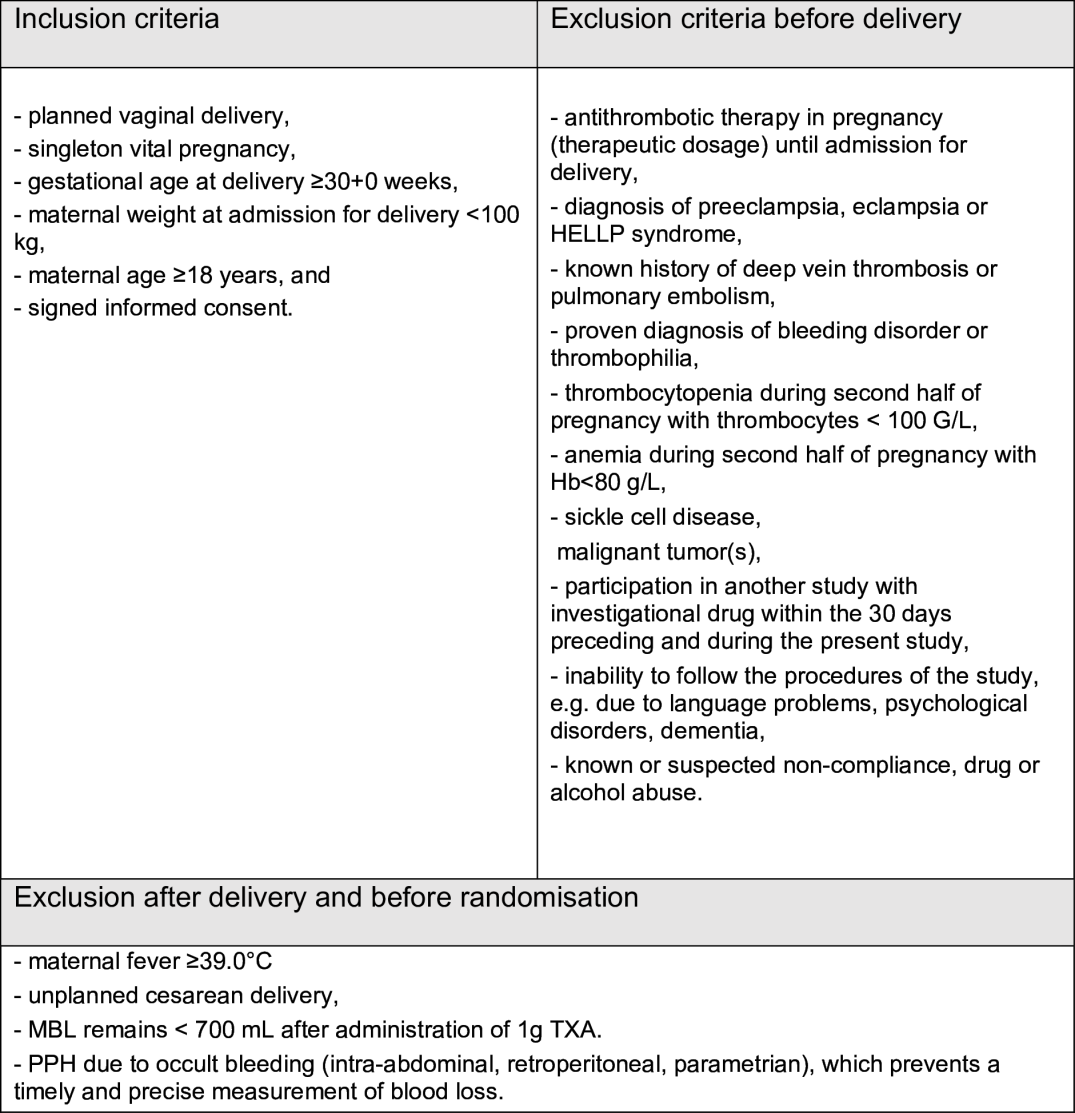
Eligibility criteria, before and after delivery. Hb, haemoglobin; MBL, measured blood loss; PPH, postpartum haemorrhage; TXA, tranexamic acid.

Adult, pregnant women, with planned vaginal delivery after 30 weeks of pregnancy and a maternal weight of less than 100 kg (at admission to delivery) are included after they provided written informed consent. The model consent form is provided in online supplemental material. Only vital, singleton pregnancies are considered for preliminary inclusion. The following exclusion criteria apply: antithrombotic therapy (therapeutic dosage) during pregnancy for any indication; pre-eclampsia, eclampsia or haemolysis, elevated liver enzymes, and low platelets (HELLP) syndrome; known history of deep vein thrombosis or pulmonary embolism; known diagnosis of bleeding disorder or thrombophilia; known thrombocytopenia during the second half of pregnancy with thrombocytes<100 G/L; known anaemia during the second half of pregnancy with haemoglobin(Hb)<80 g/L; known sickle cell disease. Additional exclusion criteria are known malignant tumour(s), participation in another interventional study, suspected inability to follow the procedures of the study and non-compliance such as drug or alcohol abuse (figure 1).

The final eligibility check comes after delivery because women shall not participate in the trial if the postpartum bleeding remains below 700 mL, if an unplanned caesarean section is to be done or if the woman developed fever≥39°C between admission and delivery. Identifying patients with fever shall exclude women with clinical chorioamnionitis. Information and consent procedures are preferably conducted during routine pregnancy visits at the hospital. However, women who enter the hospital shortly before delivery, but who are not in distress due to labour, receive an abbreviated version of patient information and go through a shortened informed consent process before delivery. Additional information is given after delivery, and women’s willingness to participate is then reconfirmed; if not, data collected may not be used.

### Ethical considerations

The study is carried out in accordance with the protocol and with principles enunciated in the current version of the Declaration of Helsinki, the guidelines of Good Clinical Practice issued by International Council for Harmonisatio (ICH), the Swiss Law and Swiss regulatory authority’s requirements. Ethics approval was granted by the central ethics committee (Kantonale Ethikkommission Zürich/Switzerland) on 16 June 2024, reference number: BASEC 2024-00374.

### Randomisation

Patients are randomised to receive FXIII in addition to the standard-of-care therapy or standard-of-care therapy only in a ratio of 1:1. Randomisation occurs after delivery if the patient has lost 700 mL of blood and bleeding is continuing despite TXA. Randomisation envelopes are used to allocate the patients to the treatment or control groups. Opening the envelope with the lowest consecutive number available assigns the patient to the respective group.

### Study treatments and management of PPH

Patients are closely monitored for bleeding after delivery. If postpartum bleeding passes 300 mL (as described below in detail), a blood collection bag is installed underneath the woman’s pelvis in order to ensure timely and accurate blood loss measurement during the postpartum and study period. If blood loss reaches 500 mL and bleeding continues, all women receive 1 g of TXA, according to the actual standard of care. The woman becomes an actual study participant after the blood loss passes 700 mL with ongoing bleeding. This is the time of randomisation and administration of FXIII (fibrogammin) to the women in the intervention group versus nothing in the control group. Throughout the entire study period, all women receive equivalent obstetric-related treatments irrespective of the assigned group (both control and intervention groups). Obstetric treatments, such as treatment of uterine atony, surgical treatment of trauma-derived bleeding and removal of retained placental tissue, are based on the primary cause of PPH and follow the hospital’s standard-of-care practice.

**Figure 2:**
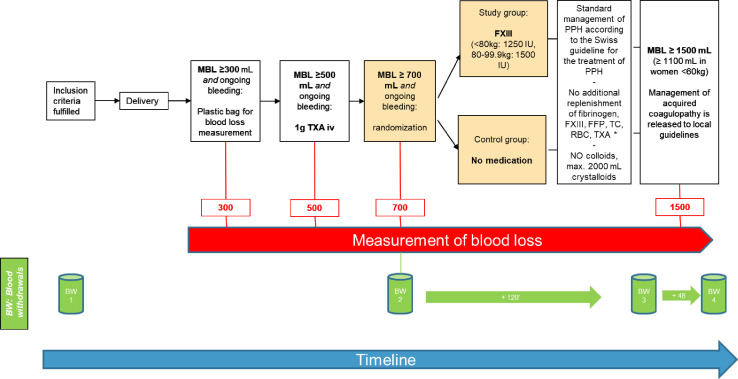
Trial flow. Women who signed the informed consent are monitored for blood loss right after delivery. After blood loss of 300 mL and ongoing bleeding: blood collection bag for quantitative measurement of blood loss is installed. After blood loss of 500 mL and ongoing bleeding: 1 g of tranexamic acid (TXA) is administered. After blood loss of 700 mL and ongoing bleeding: randomisation with allocation to study or control group. Volume and coagulation management follows a standardised procedure until measured blood loss (MBL) of 1500 mL (or 1100 mL for women<60 kg); after that threshold, coagulation management is released to local practice. Blood withdrawals (BWs) before, during and 48 hours after postpartum haemorrhage (PPH) treatment are applicable to a subgroup of patients only. FXIII, factor XIII; IU, International Units; iv, intravenous

The women in the intervention group receive either 1250 International Units (IU) of FXIII (body weight of less than 80 kg) or 1500 IU of FXIII (body weight from 80 to 99.9 kg), based on the women’s weight before delivery. FXIII is administered intravenously according to the manufacturer’s instructions. If the woman is assigned to the control group, there will be no treatment in addition to obstetric routine practice for the treatment of PPH (figure 2).

For the benefit of standardising the clinical procedures while guaranteeing the patient’s safety at all times, the participating sites are asked to administer no additional coagulation factors (such as fibrinogen, FXIII, rFVIIa), blood products (such as fresh frozen plasma, red blood cell or thrombocyte concentrates) or a second round of TXA. Regarding volume management, no colloids shall be administered, and administration of crystalloids should be restricted to 2000 mL until the blood loss has reached 1500 mL (or 1100 mL in women with a body weight of less than 60 kg). The restrictions can be bypassed at any time by a so-called safety net, which allows deviation from the restrictions above, if a laboratory parameter or point-of-care testing indicates a significant lack of one of the measured parameters, bleeding continues and the patient life is deemed in imminent danger. The safety net values are defined as fibrinogen<2.0 g/L, FXIII<60%, platelets<50 G/LHb<70 g/L or suspect point-of-care testing (ROTEM: MCF A10Fibtem≤ 6 mm; or TEG functional fibrinogen:≤10 mm).

Once the measured blood loss (MBL) reaches 1500 mL (or 1100 mL if the woman is below 60 kg), the coagulation and volume management are released to standard local practice.

### Primary outcome: MBL (in mL, within 24 hours after delivery)

Accurate and continuous blood loss measurement is a prerequisite for the analysis of the primary outcome; therefore, the SWIFT sites use a previously validated quantitative technique of real-time blood loss measurement with a proven correlation between blood loss and postpartum Hb reduction.^24^ The participating sites receive standardised blood collection bags as well as working instructions (instructions in written form and a video tutorial, please see video in the online supplemental file 2) for the purpose of achieving the maximum possible accuracy, comparability and consistency in measuring blood loss.

**Figure 3:**
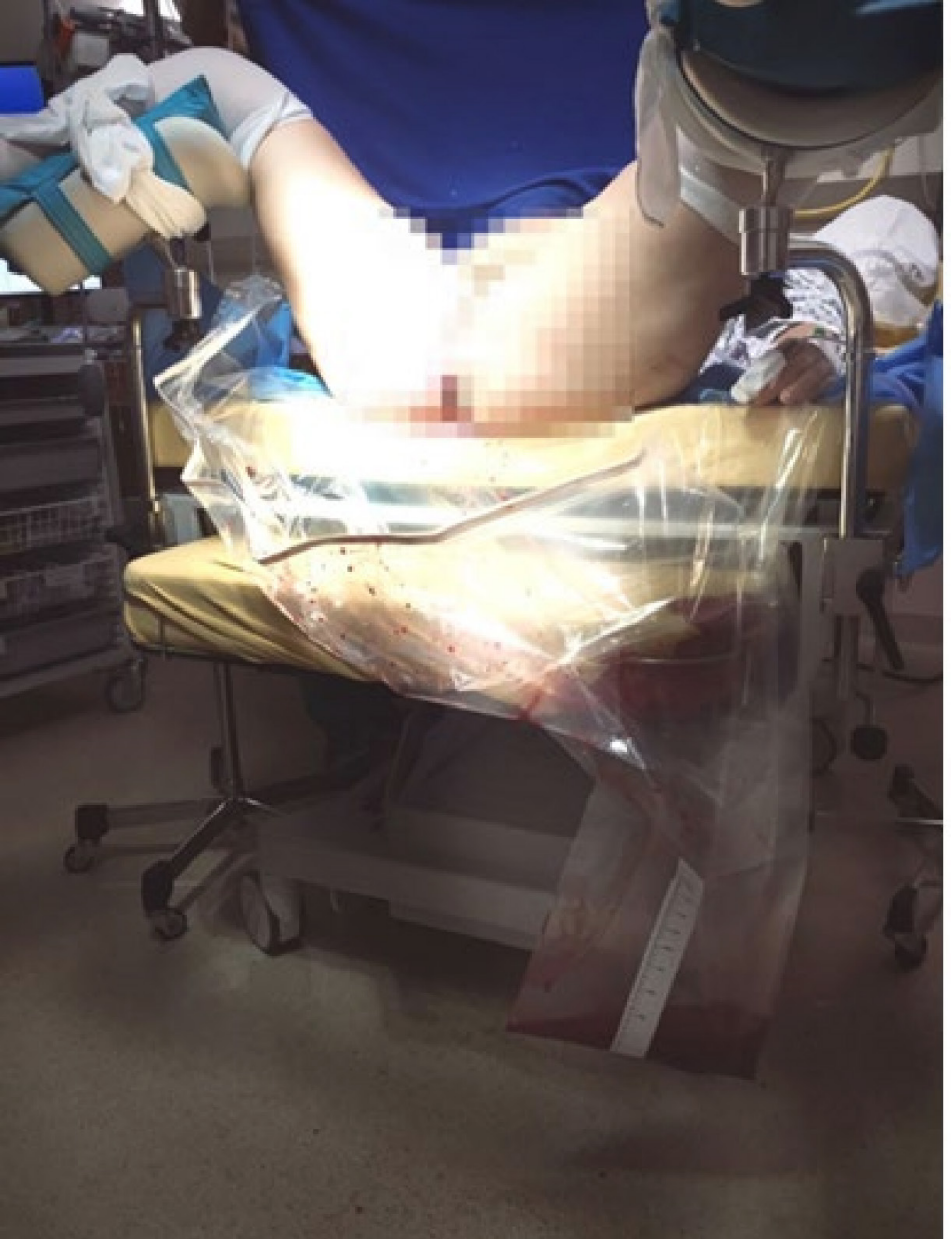
Blood collection bag with a quantitative scale for blood loss measurement in vaginal deliveries.^24^

Blood loss measurement starts immediately after delivery by placing a fresh absorbent pad underneath the woman’s pelvis. After the delivery of the baby, any additional vaginal fluid is blood and not amniotic fluid, which is absorbed by the pad. The pad is regularly checked, and if continued bleeding is observed, the pad is weighed on a neonatal scale in the delivery suite. If the weighed blood reaches 300 mL, a standardised blood collection bag with a quantitative scale is installed under the woman’s pelvis^24^ (figure 3).

Blood loss is continuously observed, quantified and protocolled. If the bag must be removed before the end of PPH (ie, for transfer to the operation room), the additional blood loss is documented either by visual estimation, weighing or other methods (such as collected blood from suction tubes in the operation room). Visual estimations (such as blood on the floor, in bedsheets) shall always be recorded with a range of minimum and maximum of observed blood loss, taking into account the uncertainty of mere blood loss observations without a measuring tool (above all in situations when blood is spilled on the floor, soaked bed linen, etc). Using a range helps provide a more realistic assessment of blood loss, reflecting the impossibility to determine exact values in these situations. Total postpartum blood loss at the end of acute PPH is used for the analysis of the primary endpoint and can be documented, consequently, as a range. This helps correct for the level of uncertainty during final statistical analysis by means of interval-censored outcome values and is definitely more realistic than a supposedly exact specification of a specific blood loss quantity. Secondary PPH, that is, increased blood loss needing medical care later than 24 hours after delivery, is recorded as a separate event and will not enter the primary outcome calculations.

### Secondary outcomes

Secondary outcomes will be compared between the intervention group and control group; time point of variable assessment will be 48 (range 36–60) hours postpartum if not stated otherwise. Composite of adverse, PPH-related maternal include MBL≥2000 mL (within 24 hours), admission to intensive care unit, blood transfusion, need for embolisation of the pelvic arteries, laparotomy with surgical measures such as compression sutures or ligatures, or hysterectomy during hospitalisation. Furthermore, changes in haematological standard values from before delivery to 48 hours after delivery such as Hb, leucocytes and thrombocyte count are assessed. (Blood samples for prepartum analysis are taken at admission for delivery, and blood samples for postpartum analysis are taken 48 hours (range 30–60) after delivery.) In specific centres, the association of antepartum and postpartum laboratory parameters with PPH and the dynamics of coagulation parameters during the course of PPH and their effect on MBL is analysed.

Analysis of differential treatment effects by means of a data-driven approach for the identification of relevant subgroups is performed.^25^

The total medical hospital costs per patient, taken from the hospital’s internal billing system, from time of admission until hospital discharge at the end of the hospital stay, will provide a first, simplified impression on the costs generated by FXIII treatment and potential savings related to fewer and/or milder adverse outcomes.

### Patient and public involvement

Given the increasing importance of patients and public involvement in research, we involved the service of the Swiss Patient Organization for the review of the patient information sheet. PPH and the management thereof often lead to stressful situations, and the woman’s feeling could be disregarded while providing emergency treatment affecting her psychosocial and emotional well-being. Recent publications point to the risk of post-traumatic stress disorder and depression in women who had a primary PPH.^26 27^ Therefore, a subgroup of patients in the early phase of the trial is asked for voluntary patient survey by providing feedback on the atmosphere in the delivery room during treatment of PPH in the main study centre. A questionnaire, designed in collaboration with the psychology department of the University of Zurich, asks for the woman’s personal sensations such as safety, anxiety, distress or insecurity. The survey is distributed and collected at discharge from the hospital, in general, 3–5 days after delivery. Once the feedback of the subgroup has been analysed, potentially negative behavioural patterns of the staff can be addressed to increase the well-being of future patients in identical situations.

### Patient follow-up

6–9 weeks after the delivery, women are contacted in order to assess the occurrence of adverse events, particularly thromboembolic events. Also, the number of women who breastfeed their babies after PPH will be recorded since PPH and its related adverse outcomes may affect the women’s ability and/or willingness to breastfeed their newborn child.^28^

### Statistical considerations

For the primary analysis, the blood loss distributions of the intervention and control group are compared by means of the proportional OR in a proportional odds model allowing continuous and interval-censored MBLs. The model corresponds to a series of binary logistic regression models for all possible cut-off points in MBL featuring a common OR treatment effect parameter. A corresponding estimate, along with a two-sided 95% CI and two-sided p value against the null hypothesis OR=1, is obtained from the Continuous Outcome Logistic Regression (Colr) model.^29 30^ Interval censoring and right-censoring in the primary endpoint are explicitly allowed and handled by the corresponding likelihood in the Colr model.

The null hypothesis of no treatment effect (OR=1) of replenishment of coagulation factor XIII in women with PPH (blood loss≥700 mL) on MBL is formally rejected when the p value is <0.05.

### Determination of sample size

Previous research quantified the effect of coagulation factor XIII by a partial OR of 1.011 (95% CI 1.006 to 1.015).^21^ This means that increasing factor XIII by one unit increases the odds of losing less than a specific amount of blood by 1.1% for constant values of prepartum Hb, prepartum fibrinogen and prepartum factor II. The marginal OR (in a model only conditioning on coagulation factor XIII, but not on prepartum Hb, prepartum fibrinogen and prepartum factor II) is almost identical to the partial OR (1.01094 vs 1.01091). It is assumed that replenishment of coagulation factor XIII leads to an increase of 30 units, which is equivalent to an expected treatment effect of OR=1.01091^30^ = 1.38491.

Sample size was calculated on the subset of the data,^21^ which suggested that the primary analysis aiming at the demonstration of a positive treatment effect (with type I error of 5% and type II error of 20%) would reasonably require 2×494 women with PPH≥700 mL to be randomised in this trial. At this sample size, the power to detect a significant difference in the occurrence of major PPH (defined as binary event MBL>1000 mL) would be about 0.64; this explains why, in the case of a cut-off variable as the primary outcome, the required number of patients would be significantly higher compared with the continuous outcome logistic regression model without achieving any additional scientific benefit.

### Current trial status (updated 27 March 2025)

More than 800 patients have already been screened, and 147 patients have been randomised since the official start of recruitment on 9 July 2024 in Zurich.

### Ethics and dissemination

The findings of this study will be published open-access in appropriate medical journals, as well as presented at national and international conferences. The data-sharing plan follows the Findable, Accessible, Interoperable and Reusable (FAIR) principles^31^ for scientific data and is provided as online supplemental material.

Data collection, maintenance and management are supported by the use of electronic case report forms, which are generated by members of the research team with support from the Data Management Department of the Clinical Trials Centre, University Hospital Zurich, Switzerland (CTC Zurich). Appropriate coded identification is used to enter participant data into the database. The Electronic Data Capture software REDCap (www.project--‐redcap.org) is used for data processing and management. We use a risk-based monitoring approach based on the categorisation as a low-risk trial.

## Discussion

This is the first randomised clinical trial investigating the use of FXIII in patients with PPH in a multicentre, obstetric setting. Furthermore, no previous report has analysed the early use of FXIII during PPH and its possible effect on reducing blood loss and, consequently, reducing the number and severity of PPH-related adverse maternal outcomes. The rising numbers of PPH-related maternal severe morbidity and mortality request new ways of thinking; even more so considering that the coagulation factor that is in many places probably given first and most frequently (fibrinogen) has not been shown to have any positive effect in three out of three randomised controlled trials in women with PPH.

The standardised blood loss measurement is a key element of the present trial. Blood loss is estimated in current routine practice most of the time, whereas this study will provide standardised, measured results using blood collection bags already at a weighed blood loss of 300 mL. When measurement is not possible, the range given for estimated blood loss provides an indication for the accuracy of the data, which is taken into consideration during the statistical analysis. Another important aspect is the defined coagulation and volume management in all participating centres until an MBL of 1500 mL; only after that, coagulation management is released to the centre’s local practice, which, however, is obviously uniform for women in the intervention as well as the control group within the centre.

Blood samples taken before, during and after the treatment of PPH in two study centres will result in a unique biological and clinical database, which will act as a hypothesis-generating motor for many studies to come, all of these aiming at improved coagulation management during the treatment of PPH. Finally, yet importantly, the patient survey will potentially improve the emotional care for patients in the future by learning from their expectations and experiences in emergency situations.

## Supplementary material

10.1136/bmjopen-2025-100262 online supplemental file 1

10.1136/bmjopen-2025-100262 online supplemental file 2

10.1136/bmjopen-2025-100262 online supplemental file 3
