# Supplementary material for: Effects of early factor XIII replacement in postpartum hae morrhage: study protocol for a multicentre, open-label, randomised, controlled, investigator-initiated trial
Source: BMJ Open. 2025 May 8;15(5):e100262. doi: 10.1136/bmjopen-2025-100262 (PMC12067823; doi:10.1136/bmjopen-2025-100262)
Supplement: online supplemental file 1 [file bmjopen-15-5-s001.doc]

Data Sharing Plan for Protocol: The effects of early factor XIII replacement in postpartum haemorrhage: multi-centre, open label, randomized, controlled, investigator-initiated trial

This document aims to ensure computational reproducibility of all published research findings obtained from data collected in this trial. Each publication will be accompanied by a dedicated compendium containing deidentified individual patient data necessary to independently reproduce the analyses presented in the corresponding publication. Such a compendium also contains information about the computer code that was used to generate figures, tables, and other statistical output.

| Will individual participant data be  available (including data dictionaries)? | Yes |
| --- | --- |
| What data in particular will be shared? | Deidentified individual participant data containing variables that underlie the results reported in the corresponding publication (text, tables, figures, and appendices). |
| What other documents will be available? | Study protocol, data analysis plan, data dictionary, analysis code, description of computational environment |
| When will data be available? | At the time of publication. |
| With whom? | Access will be provided without limitations. |
| For what types of analyses? | Data will be provided to allow independent computational reproducibility of already published results. |
| By what mechanism will data be made  available? | Distribution via a data repository following FAIR principles (such as zenodo.org). |
