# Supplementary material for: Effects of early factor XIII replacement in postpartum hae morrhage: study protocol for a multicentre, open-label, randomised, controlled, investigator-initiated trial
Source: BMJ Open. 2025 May 8;15(5):e100262. doi: 10.1136/bmjopen-2025-100262 (PMC12067823; doi:10.1136/bmjopen-2025-100262)
Supplement: online supplemental file 3 [file bmjopen-15-5-s003.docx]

**Request to participate in clinical research**

**Study Title**: **Early factor XIII replacement in postpartum hemorrhage: multi-center, randomized, controlled, investigator-initiated trial**

**Layman's title: Influence of a blood coagulation factor 13 (factor 13) on bleeding after childbirth**

Dear Madam,

We would like to inform you about our research project on the effect of a blood coagulation factor (called factor 13) on major blood loss after childbirth and ask you whether you are willing to participate.

This research is called clinical study. We are analysing if the treatment with the blood coagulation factor 13 (as short infusion) has an effect on major blood loss right after childbirth. A blood coagulation factor is a necessary substance to blood clotting. You are pregnant and d your child will be born soon. In the event of a major bleeding, treatment has to occur rapidly, and therefore we ask already now about your willingness to participate in this study. The final inclusion to the study, however, will only happen in case of a severe bleeding (more than 700ml) after childbirth. In the absence of bleeding, you not be included into the study but you can participate in a supplemental research project by providing additional blood samples for blood coagulation studies and/or for a biobank.

The participation is voluntary. The study information sheet shall provide all necessary information for you to decide on your participation. Furthermore, we invite you to ask any questions and to discuss the project with your investigator. That’s how we call the doctors, who are responsible for the study and who will take care of you during the study. If you want to participate, please sign the consent form at the end of this document. With your signature, you confirm to have read and understood the study information. If you have any additional questions, please ask the investigator.

The study information and consent has four parts

Part 1 Brief information about the most important facts
Part 2 Additional information about the study
Part 3 Data protection and insurance coverage
Part 4 Declaration of consent

Part 1 offers an overview of the study, while Part 2 explains the background and the procedures in detail. Data protection and insurance coverage are listed in Part 3. Your signature at the end of the document (Part 4) confirms that you have understood and you are willing to participate in the study.

Prof. Dr. med. Christian Haslinger, University Hospital Zurich, is the Sponsor-Investigator of the study. Sponsor-Investigator means that this study is done without the participation of industry. The study has been reviewed and is also financed by Swiss National Science Foundation.

Your contact during the study:

Name
Address Phone (24-hour availability)

E-Mail

**Part 1:
Brief information about the most important facts**

Vor allem ab Phase-3-Studien (IMP) oder konfirmatorischen MD-Studien.

**Why we are conducting this research project?**

Blood loss after childbirth is rather common whereas severe bleeding after childbirth happens rarely and unexpectedly. Current treatment options are medications, blood coagulation factors and blood transfusion together with measures taken by the obstetricians. All these serve to stop the bleeding and to replace the blood loss. Previous research showed a strong correlation between the blood coagulation factor 13 and the blood loss after delivery. The study is a logical next step in order to find out if an early administration of blood coagulation factor 13 as short, intra-venous infusion may be effective in reducing the blood loss and thereby preventing complications linked to bleeding.

In a supplemental project, we are investigating the importance of coagulation factors before and after birth. If you want to participate, we need a separate consent for the collection of your blood samples and for the further use of your health-related data and blood samples for research purposes (see Part 4).

Section 4 provides additional background information.

**What will happen if I decide to take part?**

If you are bleeding (more than 700 ml) after childbirth:

You will be randomly (by chance) assigned to the treatment or the control group. The chance of receiving factor 13 is 50%.
The treatment group receives factor 13 in addition to the standard treatments for bleedings. The control group is treated according to the hospital’s standard protocol during bleedings.

Which data are we collecting?

We will measure the total amount of blood using an absorbent pad and a blood collection bag

We will examine you and ask questions related to your health after the bleeding and before you are discharged from the hospital.

We will contact you 6 to 9 weeks after childbirth (Phone call, duration approx. 15 minutes)

In Section 5 you will learn more about procedures in the study.

**RISK AND BENEFIT**

**Benefit**

We hope to positively influence the progression of bleeding by giving blood coagulation factor 13. If you’re part of the control group, there is no direct benefit. Your participation is meaningful for research and may help future patients.

**Risk**

The study medication is authorised by Swissmedic, the Swiss Agency for Therapeutic Products, under the name of Fibrogammin. The following risks and side effects are known and described in the package leaflet:

- Allergic reaction with severe hypersensitivity affecting the whole of the body (rare, 1 to 10 women in 10,000 or 0.01%-0.1%)

- Formation of proteins that inhibit factor 13 during a new treatment (very rare in less than 1 woman in 10,000, less than 0.01%)

- Rise in temperature (rare, 1 to 10 women in 10,000 or 0.01%-0.1%).

During treatment, you will be under medical supervision so that immediate action can be taken if side effects occur. In general, we like to point out that the mediation is has a favourable risk profile. Also the medication is already known and the research consists of administering a known product at an earlier point in, instead of waiting until a high blood loss has occurred.

According to the package leaflet, Fibrogammin may be used during breastfeeding, since it is assumed that it will not pass into breast milk. Its use shortly after childbirth has no influence on breastfeeding.

Written consent if information is provided shortly before delivery:

My participation is voluntary

| Place, date | Surname and first name of the participant  Signature |
| --- | --- |

| Place, date | Surname and first name investigator  Signature |
| --- | --- |

**Part 2
Additional information on the study**

**SCIENTIFIC BACKGROUND**

**Background**

The number of women with severe and critical blood loss after childbirth is increasing worldwide, leading to a corresponding increase in complications linked to bleeding. The bleeding may have various causes: e.g. a piece of the placenta has remained in the uterus or the contraction of the uterus are insufficient after delivery. The treatment of the underlying causes of bleeding is independent of the study and corresponds to the regular clinical procedure. Those procedures will not change, if you participate in the study or not. In addition to the treatment of the causes of bleeding, a normal coagulation “or blood clotting” is key for stopping the bleeding. We see this in daily life, for example after a small cut, when we apply pressure to slow down the bleeding first and then the natural blood clotting prevents a renewal of the bleeding. Earlier research showed a strong correlation between factor 13 and the postpartum blood loss, therefore we investigate if factor 13 (given by short intravenous perfusion) at an early stage (r earlier than usual) may help to reduce the bleeding and also prevent complications due to blood loss. Our assumption is that the administration of factor 13 before major blood loss will reduce the number of women who suffer from severe blood loss in the end.

In an additional project, we are investigating the importance of coagulation factors before and after birth by taking a maximum of 4 blood samples (6 ml each, total 24 ml) at various times shortly before delivery until 2 days after delivery. In case you are not bleeding after delivery, we will examine one prenatal blood sample and two postnatal blood samples (t 2 hours and t 2 days after birth). We need your separate consent for the collection of these blood samples and the further use of your health-related data and your samples (see Part 4).

Factor 13, a protein normally produced by the liver is participates in normal coagulation. We know that there is a decrease in the body's own Factor 13 in the last trimester of pregnancy and this might be linked to the occurrence of major bleeding. In Switzerland, factor 13 (Fibrogammin) is approved as a medication for the treatment of blood clotting disorders and is used during major surgery. Fibrogammin is a purified concentrate of blood coagulation factor 13 and is obtained from human plasma.

**Design of the study: How do we proceed?**

In order to obtain reliable results from the study, the participants are assigned by chance to the experimental group or the control group. This is called randomisation.

- Group 1 (experimental group) receives the study medication (Fibrogammin) as intravenous infusion in addition to the hospital’s standard treatment.

- Group 2 (control group) will only receive the hospital’s standard treatment.

The study is a so-called open study. "Open" means that the investigators involved in conducting the study know, which group the participants have been allocated to.

**Regulation on scientific research with human subjects**

We are performing this study as required by the Swiss laws (human research act, data protection acts). Furthermore, we obey to all internationally recognized regulations. The competent ethics committee has reviewed and approved the study.

This is a national study with 988 participants. A description of this study can also be found on the website of the Federal office of Public Health at [www.kofam.ch](http://www.kofam.ch) under BASEC number 2024-00374

**COURSE OF THE STUDY**

**What do you have to do if you take part?**

Participation in the study is voluntary and takes 6-9 weeks from treatment to the final visit. You must adhere to the schedule (see **Chapter 5.2**) and all instructions given by your investigator. However, the only study intervention is the administration of factor 13, which takes only a few minutes. We request no additional visits after your hospital stay, except for a phone call (approx.15 minutes)

You must inform your investigator, if your state of health changes, e.g. if you feel worse or if you have new symptoms; this also applies if you discontinue the study prematurely (see **Chapters 5.3 and 5.4**).

**Study procedures**

During the first visit, at the latest when you are admitted to the hospital, we will explain the study to you and answer your questions. Then you can make a final decision as to whether you would like to participate or not.

If you experience severe bleeding after giving birth, you will be allocated randomly to one of the two groups (see **Chapter 4.2**) and you will be treated accordingly. The study appointments are the same for both groups and the only additional study -elated appointment is a phone call (maximum 10-15 minutes). Please refer also to the table below.

What we will do in addition to examinations and questions about your health:

- We measure blood loss by placing an absorbent pad and a special collection bag under your buttocks after the delivery.

- We will ask you about your state of health, 6 to 9 weeks after childbirth. We would also like to know whether you are breastfeeding.

- Optional, only if you agree: We take 4 a maximum of 4 additional blood samples for a supplemental research project.

The schedule below shows all the appointments. The **general examinations** are marked with
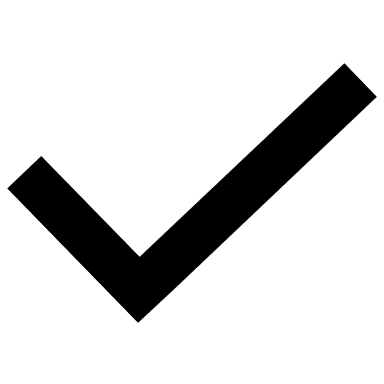
 . The **additional assessments** for the study are marked with a plus sign ( **+** ). Only those will take additional time.

| **Visit** | **0** | **1** | **2** | **3** | **4 *** |
| --- | --- | --- | --- | --- | --- |
| **When?** | **Before childbirth** | **Beginning to end of bleeding** | **2 days after childbirth** | **Hospital discharge** | **6-9 weeks after childbirth** |
| **Where?** | **During hospital stay** | | | | **By phone** |
| Information and consent | 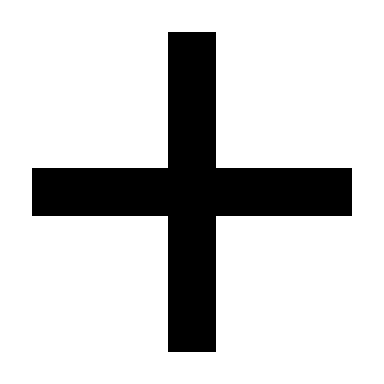 |  |  |  |  |
| Blood pressure, weight, temperature | 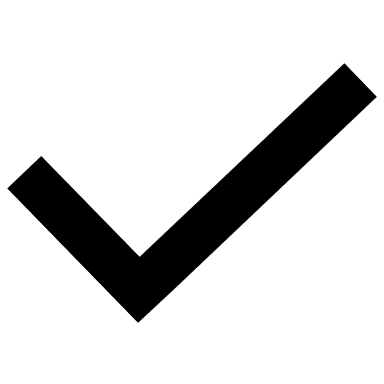 |  |  |  |  |
| Routine blood sampling | 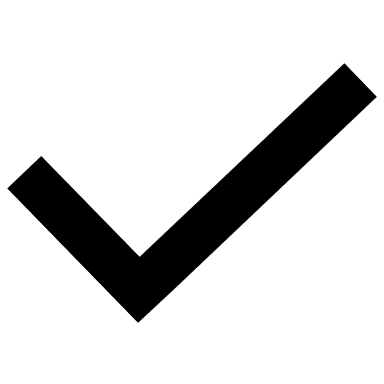 |  | 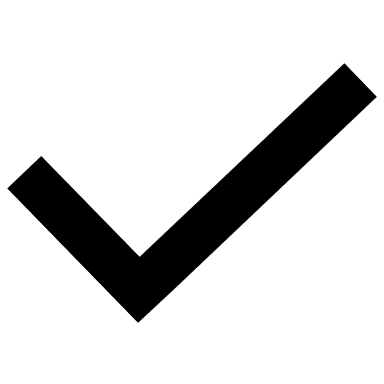 |  |  |
| Additional blood samples for research/biobank (optional) |  | 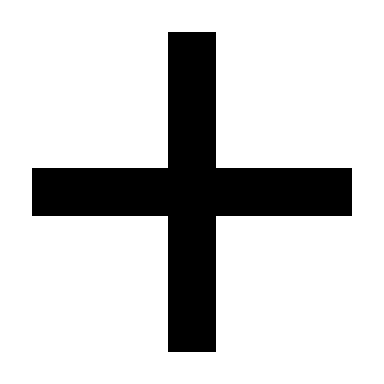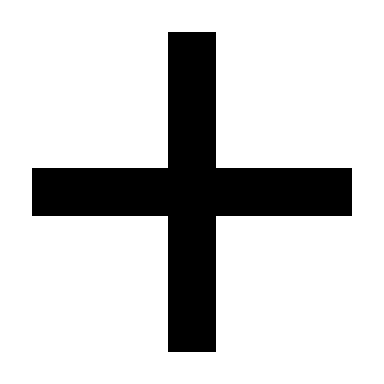at beginning of bleeding and 2 hours later |  |  |  |
| Measuring blood loss |  | 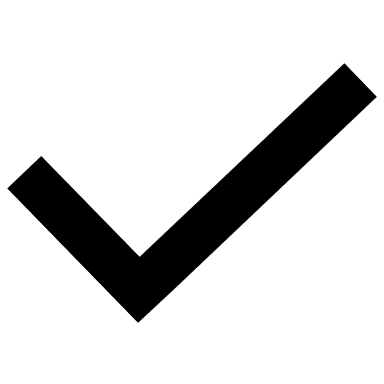 |  |  |  |
| Questions about health status | 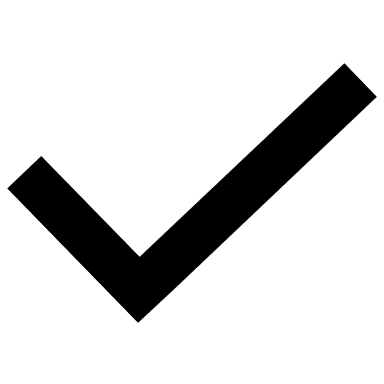 | 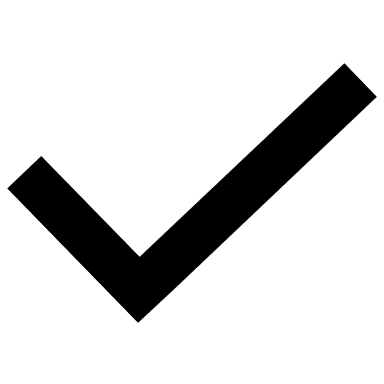 | 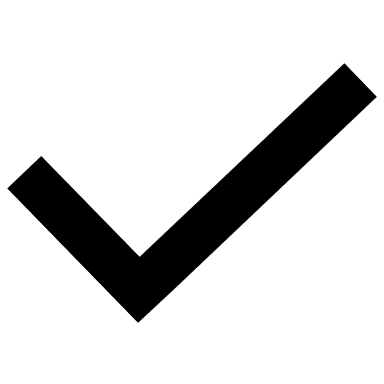 | 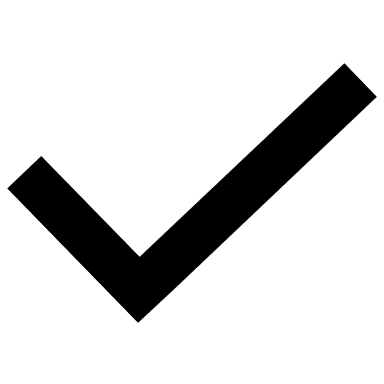 | 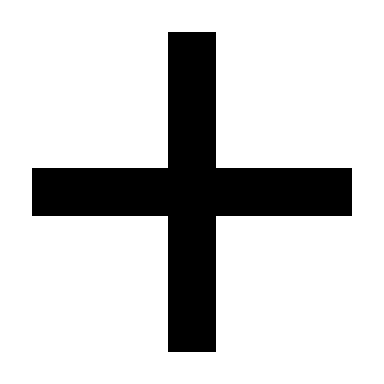 |
| Questionnaire on personal experience during bleeding |  |  |  | 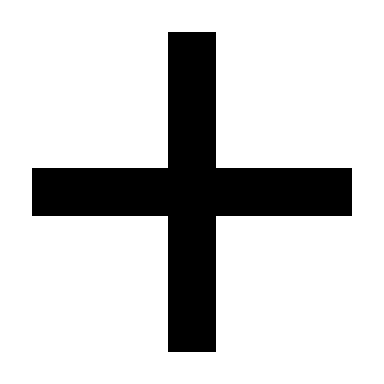 |  |
| Questions about breastfeeding |  |  |  | 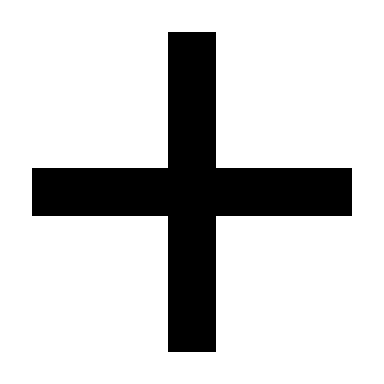 | 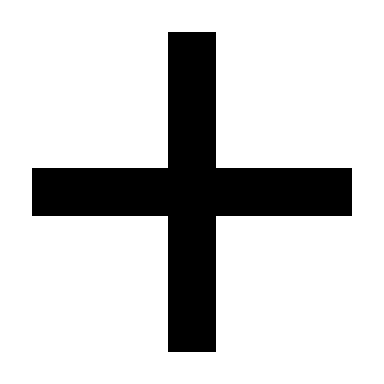 |

*This telephone call is a study appointment only.
The dates are calculated from the date of childbirth.

**When does the participation end?**

If bleeding occurs, your participation and will end after to 6-9 weeks after childbirth with the telephone appointment. You can end your participation at any time (see Chapter 5.4) without explaining. If you would like to end your participation prematurely, please speak to your investigator.

Even if your participation ends prematurely, we will continue to treat and care for you medically in the best possible way in accordance with current standards (see Chapter 5.4). We will carry out a final examination for your safety.

If you discontinue the study, we ask you to continue to inform your investigator if your state of health changes, e.g. if you feel worse or if you have new symptoms. We will analyse the data and samples collected up to that point, and if you agreed separately we will store them in a biobank for further use. Your data and samples remain encrypted.

We may have to ask you to end the study prematurely, for example, if you have an unplanned caesarean section or if you have a fever at the time of giving birth or if you are bleeding less than 700 ml (what we sincerely wish for).

**What happens if you do not want to participate?**

Even if you do not participate in this study, we will treat and care for you medically in the best possible way according to current standards. Underlying causes of the bleeding will be treated according to hospital standards and factor 13, if at all, may be used at a later point.

**RISKS, BURDENS AND SIDE EFFECT**

**Risks and burdens that may occur**

As with any medical treatment, there are risks and burdens associated with participating in this study. Some risks are known, while others may still be unknown. This uncertainty is not unusual in the study environment. You will find a list of the most common and most serious risks in Chapter 6.2. Many side effects can be treated medically. We will inform you of any new findings on risks and side effects during the study. The study medication is authorised by Swissmedic, the Swiss Agency for Therapeutic Products. Also, it has been used for many years in obstetrics, gynaecology as well as other therapeutic areas, where bleedings occur and it showed a favourable rate of side effects.

**Most common and most serious side effects**

These are the most common and most serious side effects that we are already aware of.

We use the following descriptions:

| rare | We find the side effect in 1 to 10 people out of 10,000 (0.01%-0.1%). |
| --- | --- |
| very rare | We find the side effect in less than 1 person in 10,000 (less than 0.01%). |

A rare but dangerous side effect is

- Allergic reaction. A severe allergic reaction is characterised by a rapid pulse, breathing problems and swelling of the face and the whole body.

- Rise in temperature

A very rare but dangerous side effect:

- Formation of proteins that inhibit factor 13.

**Risks and burdens due to the additional project**

We will take either 3 or 4 additional, study-related blood samples of 6 mL each. (4 blood samples if you have a bleeding, and otherwise only 3 blood samples). This may be uncomfortable for you and may cause bruising or swelling at the puncture site, but the additional amount of blood taken does not cause any harm. In rare cases, an infection may occur at the injection site. Wherever possible, we try to take the study-related blood samples at the same time as the routine blood samples in order to reduce the burden.

**Funding and compensation**

This study is initiated by Prof. Christian Haslinger, the Department of Obstetrics, University Hospital Zurich. The funding is provided by the Swiss Science Foundation, meaning that there is no funding needed by industry.

The researchers have no financial advantage from the conduct of the study. Also, you will not receive any compensation for your participation. There are no costs for you or your health insurance.

**Results**

The investigator will inform you during the course of the study about any relevant new results and findings. There may be incidental findings. Incidental findings are "accompanying results" that are not intended, for example, results from the blood analysis. We will inform you if these incidental findings are relevant to your health. If you do not wish to be informed, please discuss this with your investigator.

The study will also generate overall results from the data of all participants. These results do not affect you and your health directly. However, your investigator will provide a summary of the overall results at the end of the study, if you wish.

**Part 3
Confidentiality and Insurance**

**Protection of Data and Samples**

We protect your data (e.g. information such as blood pressure and pulse from your medical records) and your samples (e.g. your blood samples). Switzerland has strict legal regulations for the protection of data and samples.

The Swiss Data Protection Act gives you the right to information, correction and receipt of your data that is collected, processed and forwarded as part of the study. These rights cannot always be guaranteed in exceptional cases due to other legal or regulatory requirements. If you have any questions, please contact your investigator.

**Data and sample encryption**

Each study generates data from the examinations (e.g. blood values, blood loss after birth). Documentation of the data is usually done electronically in large tables, the so-called "case report forms ". The data is documented in encrypted form. "Encrypted" means that personal information is stored separately from the examination results. For this purpose, there is a list that identifies each person with a unique code. For example, your name, date of birth or place of residence are not directly included in the case report form. The list remains at the hospital for a period of 10 years. Nobody else receives this list.

If we transfer data to the sponsor-investigator or to specialists or organisations for additional assessments, the data is always encrypted and personal data is protected. This also applies if the data is transferred abroad.

**Safe handling of data and samples during the study**

The investigator-sponsor is responsible for the safe handling of your data and samples from this study. He is responsible for ensuring that the applicable laws, e.g. data protection laws, are complied with. This also applies if (encrypted) data for studies are sent to countries where data protection laws are less favourable.

In this study, your data is collected and transmitted electronically. The data is stored in encrypted form on a server in Switzerland. Nevertheless, there is always a small residual risk that third parties may access your personal data (e.g. risk of "hacking").

It may become important that your general practitioner or other treating physicians share data with the investigator. You agree to this by signing the consent form at the end of this document.

**Safe handling of data and samples after the study**

The sponsor-investigator remains responsible for the secure handling of your data and samples even after the end of the study. The law stipulates that all study documents, e.g. the data collection forms, must be kept for at least 10 years.

If you agree, we collect additional blood samples and keep them encrypted in a safe place for at least 10 years. They may be used for future analysis (🡪 section 9.4). Such a collection of encrypted samples is called” biobank “. Biobanks are regulated strictly in order to safeguard information related to your samples.

At the end of this long period, study data remains encrypted. Health-relevant data from your medical history, including from this study, are and will always remain accessible to those treating you.

Once a study has been completed, the results are usually published in scientific journals and the results are evaluated by other specialists. Your encrypted data will be forwarded to these specialists.

**Collection and further use of your data and samples for research (supplemental project and biobank)**

Your data and samples from this study are very important for research. In a supplemental research project, we would like to analyse data and blood samples to gain further knowledge on coagulation. These samples (or remnants thereof) can possibly also be used for future research. We need your separate consent for the collection of the additional blood samples, for the use of your health-related data and for further use (in a biobank). This is voluntary. Please read the additional declaration of consent at the end of the document carefully. Even if you do not consent, you can still take part in the SWIFT study. Further research projects with the biological material you have provided and the associated data may only be carried out with the approval of the responsible ethics committee.

**Rights during inspections**

The conduct of this study may be reviewed by authorities such as the responsible ethics committee or Swissmedic. The sponsor investigator must also carry out such reviews to ensure quality and results.

For this purpose, a small number of specially trained persons are given access to your personal data and medical history. The data is not encrypted for this review. The people who see your unencrypted data are subject to a duty of confidentiality.

As a study participant, you have the right to view your data at any time.

**Liability**

You are insured in case of damage that would have occurred due to your participation in the study. The procedure is regulated by law. For this purpose, the University Hospital Zurich has taken out an insurance policy at “Zurich Versicherung”, Police N° 14.970.888. If you think you have suffered harm because of the study, please contact your investigator or the insurance company directly.

**Part 4
Informed Consent**

You will find two independent declarations of consent hereafter:

- Declaration of consent for participation in the SWIFT study.

- Declaration of consent for participation in an additional research project (with blood sampling) and for the further use and transfer of data and samples in encrypted form (biobank)

Please read this form carefully. Please ask if there is anything you do not understand or if there is anything you would like to know. Your written consent is required for participation.

**Declaration of consent for participation in the SWIFT study**

| **BASEC-Number** | 2024-00374 |
| --- | --- |
| **Study Title** | SWIFT “Early factor XIII replacement in postpartum hemorrhage: multi-center, randomized, controlled, investigator initiated trial” |
| **Title in lay language** | **Influence of a blood coagulation factor 13 (factor 13) on bleeding after childbirth** |
| **Sponsor** | Prof. Dr. med. Christian Haslinger  UniversitätsSpital Zürich  Frauenklinikstrasse 10  8091 Zürich |
| **The study takes place at** |  |
| **Investigator at the study site** |  |
| **Participant:** Name and first name: Date of Birth: |  |

- I have received verbal and written information about the study from the investigator signing below.
- The investigator has explained the purpose, procedure and risks of the study and the treatment method to me.
- I am participating in this study voluntarily
- The investigator explained the standard treatments available outside of the trial.
- I have had sufficient time to make this decision. I keep the written information and receive a copy of my written informed consent.
- I can end my participation at any time. I do not have to explain why. Even if I end my participation, I will continue to receive my medical treatment. The data and samples collected up to that point will be stored and analysed as part of the study.
- If it is better for my health, the investigator can exclude me from the study at any time.
- My general practitioner as well as other treating physicians may share data from my medical records with the investigator, if relevant for the trial.
- I understand that my data will only be transferred in encrypted form. The sponsor will ensure that data protection is in accordance with Swiss standards.
- I will be informed of any results and/or incidental findings that directly affect my health. If I do not wish to be informed, I will discuss this with my investigator.
- The responsible specialists of the sponsor, the Ethics Committee and/or the Swissmedic Medicines Agency may inspect my unencrypted data for control purposes. All these persons are subject to a duty of confidentiality.
- I am aware that the University Hospital Zurich has taken an insurance. This insurance will pay if I suffer any damage - but only if the damage is directly related to the study.

| Place, date | Surname and first name of the participant (block letters)  Signature |
| --- | --- |

**Confirmation by the investigator:** I hereby confirm that I have explained the nature, significance and scope of the study to this participant. I assure to fulfil all obligations related to this study according to Swiss law. If, in the course of the study, I learn of aspects that could influence the participant’s willingness to take part in the study, I will inform her immediately.

| Place, date | Surname and first name investigator (block letters)  Signature investigator |
| --- | --- |

**Information and consent for the additional collection of biological material as part of a research project and for further use and transfer for research purposes**

Dear Madam,

We are asking you whether you would be willing to participate in an additional research project where we are investigating blood coagulation around the time of delivery. If any biological material remains after the blood coagulation tests, we would like, with your consent, to store this material in a biobank and use it for future research questions that have not yet been determined.

Although biomedical research has made great progress in recent decades, there are still many areas in which knowledge about the causes, detection and treatment of diseases can be improved for the benefit of the patients concerned. Numerous research projects in these areas can only be realized today if biological material is available. If you decide to do so, we would like to thank you very much.

In order to be able to carry out this additional research project, we would like to take a maximum of 4 blood samples (6 ml each). Your participation is voluntary. All data collected in this research project is subject to strict data protection regulations. The background, benefits, risks, your rights of withdrawal and the practical procedure for taking blood samples are explained on the previous pages (SWIFT study information).

"Further use" means that data and samples may be stored beyond the time of your participation in the study and used in encrypted form for further research projects. This means, for example, that a blood sample and corresponding laboratory values from you are statistically analysed together with a large number of other values or that samples are used for further examinations. The analysis of your samples takes place at the Center for Laboratory Medicine (ZLM) in St. Gallen. “Transfer" means that your data and samples will be passed on to other research persons or research institutions in coded form for further research projects.

| **BASEC-Number** | 2024-00374 |
| --- | --- |
| **Study Title** | SWIFT “Early factor XIII replacement in postpartum hemorrhage: multi-center, randomized, controlled, investigator initiated trial” |
| **Title lay language** | **Influence of a blood coagulation factor 13 (factor 13) on bleeding after childbirth** |
| **Responsible institution/Project Leader** | Prof. Dr. med. Christian Haslinger  UniversitätsSpital Zürich  Frauenklinikstrasse 10  8091 Zürich |
| **Participant:** Name and first name: Date of Birth: |  |

- I am taking part in this research project voluntarily and accept the content of the written information provided. I have had sufficient time to make my decision. By ticking the box below,

⬜ I agree to the transfer and storage of my encrypted data and samples in a
biobank for further research use.

- I have been informed verbally and in writing by the investigator about the purpose, the procedure, possible advantages and disadvantages as well as possible risks.
- My questions have been answered. I will keep the written information and receive a copy of my written informed consent.
- I agree that the responsible experts of the project management and the ethics committee responsible for this research project may inspect my unencrypted data for testing and control purposes, but under strict confidentiality.
- I understand that the data and samples are encrypted and the key is stored securely. The sponsor guarantees that data protection in accordance with Swiss standards will be observed.
- I can withdraw from participation at any time and without giving reasons. My further treatment is guaranteed regardless of my participation in the research project. The data and samples collected up to that point will still be used for the evaluation of the research project.
- Normally, all data and samples are analysed together. If, by chance, a result emerges that is very important for my health, I will be contacted. If I do not wish to be contacted, I will inform my investigator.
- In the case of biobank consent: The samples and data will be stored in a biobank (Biobank B_B_EST_D_, Center for Laboratory Medicine St. Gallen). They will then be available for further examinations for an indefinite period of time.
- In the case of biobank consent: After withdrawal, my data and samples will no longer be available for new research projects. Ongoing projects will still be completed.

| Place, date | Surname and first name of the participant (block letters)  Signature |
| --- | --- |

**Confirmation by the investigator:** I hereby confirm that I have explained to the participant the nature, significance and scope of the additional research project and the further use and transfer of samples and data.

| Place, date | Surname and first name investigator (block letters)  Signature investigator |
| --- | --- |
